# A comparison of the vancomycin calcium sulfate implantation versus fenestration decompression for the treatment of sclerosing osteomyelitis

**DOI:** 10.1186/s12891-021-04881-7

**Published:** 2021-11-29

**Authors:** Haotian Hua, Xinwei Wang, Jiangang Guo, Lei Zhang, Zairan Guo, Jiangfei Chen

**Affiliations:** 1 Henan University of Traditional Chinese Medicine, Zhengzhou, China; 2 Research and Treatment Center of Bone and Joint Infections, Luoyang Orthopedic Hospital of Henan Province, Orthopedics Hospital of Henan Province, Luoyang, China

**Keywords:** Vancomycin calcium sulfate implantation (VCSI), Fenestration decompression (FD), Sclerosing osteomyelitis, Pain

## Abstract

**Objective:**

To compare the clinical efficacy of vancomycin calcium sulfate implantation and fenestration decompression in the treatment of sclerosing osteomyelitis.

**Method:**

A retrospective analysis for 46 cases of sclerosing osteomyelitis

were admitted to our department between June 2010 to June 2020. Twenty-one patients were treated with fenestration decompression, twenty-five patients were treated with vancomycin calcium sulfate implantation. The postoperative hospital stay, days of drainage tube placement, visual analogue scale scores, C-reactive protein and erythrocyte sedimentation rate were compared between the two groups.

**Results:**

The visual analogue scale scores of both groups were significantly lower than before treatment (*p <* 0.05), but the difference between them was not statistically significant. Patients treated by vancomycin calcium sulfate implantation had shorter postoperative hospital stay and days of drainage tube placement compared to those treated by fenestration decompression (*p <* 0.05). C-reactive protein and erythrocyte sedimentation rate in both groups were significantly lower than before treatment, but the improvement effect of vancomycin calcium sulfate implantation was better (*p <* 0.05).

**Conclusion:**

Both treatment methods can relieve pain effectively. Compared with fenestration decompression, vancomycin calcium sulfate implantation can shorten the treatment time effectively, control the infection better.

## Background

Sclerosing osteomyelitis is a chronic inflammatory disease that occurs in the bones. The mandible is the most common site of sclerosing osteomyelitis, and the long bones of the limbs are also prone to the disease,especially the femur and tibia [1]. This type of osteomyelitis is relatively rare in clinical practice, because the pathogenic bacteria cannot be found in the local area of the lesion [2]. The specific pathogenesis of the disease is not clear, but it is generally believed to be caused by low toxicity infection of bone tissue and strong osteogenic reaction [3]. There is widespread, progressive sclerosing inflammation of the bone following bacterial infection, which will cause extensive fibrosis in the bone marrow cavity. This extensive fibrosis will hinder the blood circulation in the bone marrow cavity, and promote the proliferation, calcification, and deposition of the bone tissue under the endosteum, which will eventually cause the thickening and expansion of the cortical bone. This pathological change progresses very slowly, and the course of the disease can often reach several years. Pain is often the main symptom of sclerosing osteomyelitis, as the patient's medullary cavity is narrowed or occluded, resulting in increased pressure in the medullary cavity [4]. Symptoms such as abscesses and necrosis, which are common in other bacterial infections, are rarely seen in sclerosing osteomyelitis.

It is difficult to diagnose sclerosing osteomyelitis because it is often only manifested as pain at the site of the disease, but the inflammatory response and related laboratory test indicators are not typical [5]. Therefore, it is often necessary to differentiate from Ewing's sarcoma, syphilitic osteitis, osteitis deformans, osteoid sarcoma, and sclerosing osteosarcoma. After diagnosis, there are many treatments that can achieve results. Some clinical studies have reported that painkillers, non-steroidal anti-inflammatory drugs, and antibiotics can all relieve the pain symptoms of patients [6, 7]. However, the bone marrow cavity of patients with sclerosing osteomyelitis is occluded, which will cause poor local blood circulation, and the drug concentration in the local blood is not enough to kill bacteria. Therefore, although these conservative treatments can relieve pain symptoms temporarily, the possibility of recurrence is high. This chronic inflammation will recur when people are in poor health, so surgical treatment is necessary. FD is an effective method to treat sclerosing osteomyelitis, which is to remove the sclerosing bone and open the medullary cavity to reduce the pressure in the marrow cavity and relieve pain, then place a drainage tube for flushing to remove local residual bacteria [8]. However, this method cannot completely remove local bacteria, which will become a hidden danger for recurrence of infection in the future. In addition, bone defects created by surgical removal may lead to pathological fractures. In recent years, the local use of antibiotics has been widely used in practice. Multiple clinical studies have reported a good effect of VCSI in the treatment of chronic osteomyelitis [9, 10]. The primary objective of this retrospective study was to compare the efficiency of FD and VCSI in the treatment of sclerosing osteomyelitis.

## Methods

This retrospective case-control study was performed analyzing 46 patients with sclerosing osteomyelitis between June 2010 to June 2020 in our hospital. The study was approved by the Luoyang Orthopedic-traumatological Hospital’s ethical review committee (KY2018-001-01). Written informed consent was obtained from all patients to use their clinical data for the clinical research.

The inclusion criteria were as follows: (1) Meet the diagnostic criteria for sclerosing osteomyelitis. (2) Patients were treated in our department, and the surgery performed was FD or VCSI. (3) The patient’s case information is complete and the follow-up is not lost. The exclusion criteria were as follows: (1) patients with other serious internal diseases; (2) The patient did not review regularly and the follow-up was lost.

The patients included in this study were divided into two groups according to different treatments, vancomycin calcium sulfate implantation group and fenestration decompression group. The baseline information of the two groups was not different as shown in Table 1.

**Table 1:** Descriptive data and disease characteristics of patients

Variables	VCSI group(*n*=25)	FD group(*n*=21)	*P*-Value
Sex (Male/Female)	18/7	15/6	0.966
Mean age (years)	26 (21)	29 (21)	0.776
Location			0.976
Femur	8	7	
Tibia	14	12	
Fibula	2	1	
Humerus	1	1	
CRP	7.37 (4.735)	6.43 (3.43)	0.125
ESR	10 (19)	9 (10.5)	0.408
VAS scores	5 (2)	5 (1)	0.889
Follow-up time (month)	29 (33)	75 (19)	

*Note*: Data shown as number or median (interquartile range). *P* < 0.05, significant difference.

*VCSI* Vancomycin calcium sulfate implantation, *FD* Fenestration decompression, *CRP* C-reactive protein, *ESR* Erythrocyte sedimentation rate, *VAS* Visual analogue scale

### Surgical procedures

Preoperative treatment: bacterial culture was performed on patients with sinus passages, then select sensitive antibiotics based on the results of bacterial culture to relieve local symptoms. The purpose of this is to clear the infection more thoroughly. For patients with negative bacterial culture results or no sinus tract, antibiotics were selected empirically for antibacterial treatment. The lesion location and intraoperative lesion clearance range were determined according to X - ray, CT and MRI. In the present study, all the surgical procedures were performed by the same surgeon.

FD group: select a suitable incision to expose the lesion after anesthesia successfully, the thickened periosteum is incised, open the cortex for fenestration decompression, the hardened bone is removed completely until normal blood flow is available to the bone, then open up the medullary cavity completely and remove purulent fluid and inflammatory granulation tissue remaining in the bone marrow cavity. Take the surrounding infected tissue for pathological examination and bacterial culture. After that, rinse with hydrogen peroxide and physiological saline repeatedly. Finally, soak in iodophor for 10 minutes. A irrigation tube is placed in the upper marrow cavity. A drainage tube was placed in the lower bone marrow cavity. External fixation was performed according to the local condition of the patient. Suture the wound intermittently, and cover the wound with a sterile dressing.

**Fig. 1:**
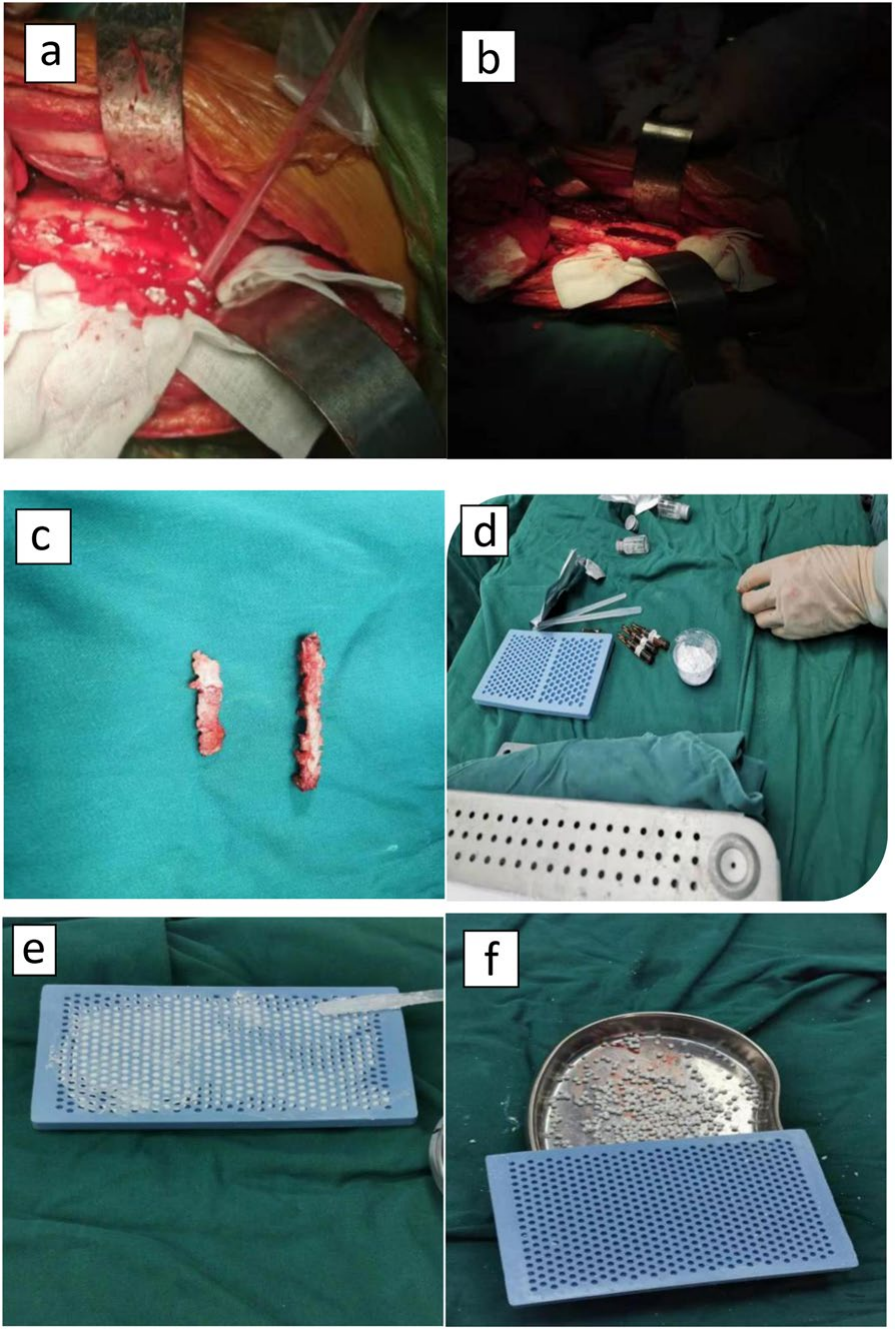
Intraoperative photographs. **a** Drill holes in the cortical bone. **b** Bone cortical fenestration and remove hardened bone. **c** Bone window. **d** Preparation of vancomycin calcium sulfate. **e** Fill in the mold. **F** Formation of particles.

VCSI group: select a suitable incision to expose the lesion after anesthesia successfully, the thickened periosteum is incised, open the cortex for fenestration decompression, the hardened bone is removed completely until normal blood flow is available to the bone, then open up the medullary cavity completely and remove the purulent fluid and inflammatory granulation tissue remaining in the bone marrow cavity. Take the surrounding infected tissue for pathological examination and bacterial culture. After that, rinse with hydrogen peroxide and physiological saline repeatedly. Finally, soak in iodophor for 10 minutes. Replace surgical drapes and gloves. The 5ml calcium sulfate powder (Biocomposites Ltd, England) was mixed with 500 mg of vancomycin powder and 1.5 ml gentamicin to make a paste. The formed paste is implanted evenly in the mold to form a 4mm×3mm ball. Then let it dry for 15 minutes. The prepared vancomycin calcium sulfate ball was placed and dried for 15 minutes and then evenly filled into the debridement site. External fixation was performed according to the local condition of the patient. Finally, place the drainage tube, suture the wound in layers, and cover the wound with a sterile dressing. Figure 1 shows an intraoperative photographs and schematic diagram of this surgical approach.

Postoperative treatment: routine anticoagulant and analgesic therapy was performed after surgery. According to the results of bacterial culture, sensitive antibiotics were injected intravenously for 2 weeks, and then switched to oral antibiotics for 4 weeks. Surgical dressing change was performed regularly, wound healing and drainage tube were observed. In the FD group, 1500 ml~2 000 ml of sensitive antibiotic solution was used for 24 h continuous infusion every day. Remove the tube when the general condition improves, the body temperature returns to normal, the pain is relieved, the drainage fluid is clear, and three times of consecutive bacterial cultures are negative.

### Result evaluation

The efficacy of VCSI and FD in the treatment of sclerosing osteomyelitis was evaluated by comparing postoperative hospital stay, days of drainage tube placement, VAS scores, relevant laboratory indicators (including C-reactive protein, and erythrocyte sedimentation rate).

### Statistical analysis

The SPSS (Version 21.0) software package (SPSS Inc, USA) was used for statistical processing. Quantitative data was presented as median and interquartile range. First, we tested normality and homogeneity of variance. If the variable met the above conditions, t test was used. If the variable did not meet the above conditions, Wilcoxon signed rank test was used. Qualitative data used chi-square test. p<0.05 means there is a significant difference between the groups.

## Results

**Fig. 2:**
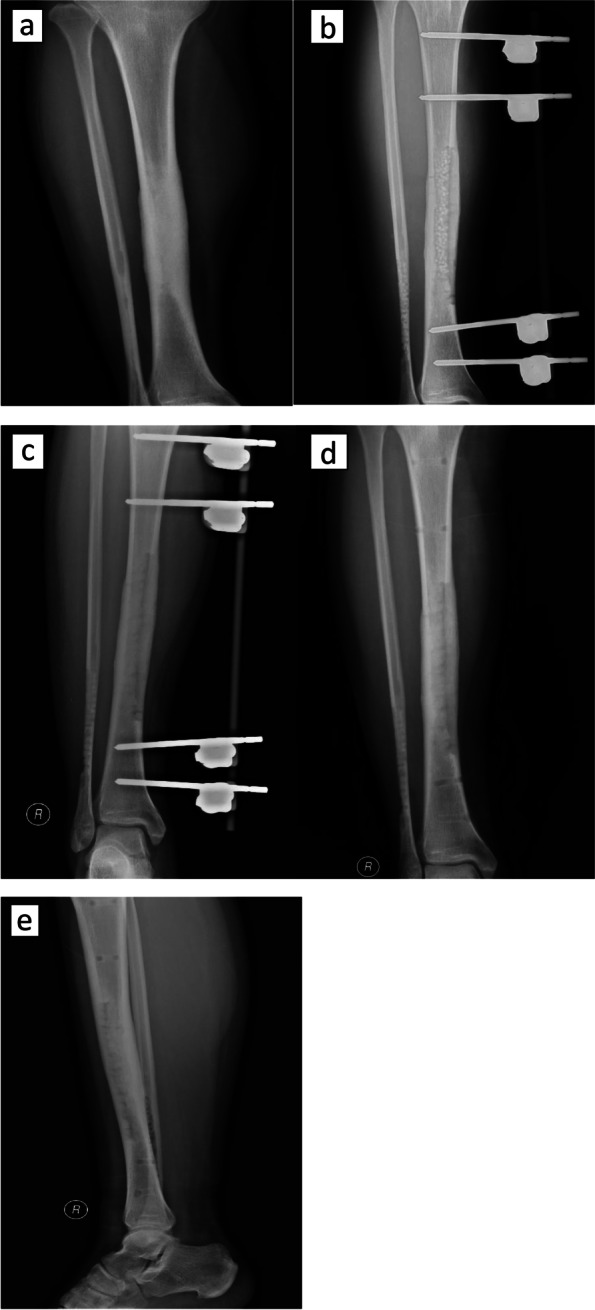
Typical case. A 31-year-old male patient presented with right leg pain that worsened at night. **a** Preoperative X-rays showed thickening and hardening of the middle and lower tibia, and narrowing of the medullary cavity. **b** Twenty days after the operation, the density of the medullary cavity of the middle and lower tibia and the lower fibula was reduced, and there was a granular high-density shadow filling. **c** Two months after the operation. **d** Five months after the operation, the external fixator was removed. **e** Seven months after the operation.

The operations of the two groups were completed successfully, and there were no serious complications such as nerve and blood vessel damage during the operation. Clinical outcomes are shown in Table 2. The median of operation time was 100 minutes (interquartile range 20) in the VCSI group, whereas that of the FD group was 80 minutes (interquartile range 30). The operation times were different significantly (*P*=0.004). The median of intraoperative blood loss was 100 ml (interquartile range 125) in the VCSI group, there was not significantly different (*p*=0.885) compared to FD group (median 100, interquartile range 125). The duration of drainage tube placement was significantly reduced (*P*=0.000) in the VCSI group (median 8, interquartile range 3), compared with the FD group (median 18, interquartile range 7). The median of postoperative hospital stay was significantly shorter (*p*=0.000) in the VCSI group (median 13, interquartile range 4) compared to that in the FD group (median 23, interquartile range 6). The VAS scores between two groups had no significant difference (*p*=0.655). The VCSI group had the advantage of significantly less (*P*=0.024) ESR (median 4, interquartile range 5) compared to the FD group (median 6, interquartile range 12.5). The VCSI group had the advantage of significantly less (*P*=0.023) CRP (median 3.10, interquartile range 1.66) compared to the FD group (median 4.79, interquartile range 4.46). One patient in the VCSI group relapsed 1 year after surgery because of cold, and was cured after another VCSI. Six patients in the FD group relapsed, two of the patients relapsed after catching a cold, and four patients had no obvious causation. Three patients were cured after another FD and three patients were cured after VCSI. A typical case is shown in the figure 2.

**Table 2:** Summary of clinical outcomes of the two groups

Observation variables	VCSI group(*n*=25)	FD group(*n*=21)	*P*-Value(95% CI)
Operation time (min)	100 (20)	80 (30)	0.004 (5.00~20.00)
Intraoperative blood loss (ml)	100 (125)	100 (125)	0.885
Duration of drainage tube placement (day)	8 (3)	18 (7)	0.000(-12.00~-8.00)
Postoperative hospital stay (day)	13 (4)	23 (6)	0.000(-12.00~-8.00)
VAS scores	1 (2)	1 (1.5)	0.655
ESR	4 (5)	6 (12.5)	0.024(-8.00~0.00)
CRP	3.10 (1.66)	4.79 (4.46)	0.023(-3.19~-0.20)

Note: Data shown as number or median (interquartile range). *P* < 0.05, significant difference

*VCSI* Vancomycin calcium sulfate implantation, *FD* Fenestration decompression, *CI* Confidence interval, *VAS* Visual analogue scale, *CRP* C-reactive protein, *ESR* Erythrocyte sedimentation rate

## Discussion

It is essential for the treatment of osteomyelitis to apply antibiotics promptly and adequately, but this method is not completely suitable for the treatment of sclerosing osteomyelitis. Because the presence of sclerotic bone affects the local blood supply greatly and makes antibiotics less effective [11]. Sclerosing osteomyelitis is a low-toxic bacterial infection, which particularity is that the inflammatory response is not obvious and the main pathological change is the thickening, and expansion of the bone cortex caused by a strong osteogenic reaction. In view of the thickening of cortical bone in sclerosing osteomyelitis and the pain caused by high pressure in the medullary cavity, FD is an effective way to solve this problem. FD of the thickened part of the cortical bone can reduce the pressure instantly in the medullary cavity and relieve the pain, but it is not enough. It is very important to open the upper and lower medullary cavity and restore the normal blood supply to the bone. Studies have shown that reaming can damage the blood vessels of the endosteum, affect the blood supply to the bone cortex, and affect the healing of the bone. However, the destruction of this kind of blood vessels will in turn promote the reconstruction of endosteal blood vessels and facilitate the formation of new bone [12–14]. Studies have reported that Staphylococcus aureus infection is the most common cause of sclerosing osteomyelitis [15]. Staphylococcus aureus will enter osteoblasts for long-term survival after infecting bone tissue, and form bacterial biofilm on the surface of the lesion. In patients with sclerosing osteomyelitis, the severe destruction of blood supply in the medullary cavity and the presence of bacterial biofilms will make systemic antibiotics less effective [16, 17]. Incomplete lesion clearance is also an important cause of osteomyelitis recurrence. Topical antibiotics can effectively solve this problem. The use of antibiotic lavage and drainage after FD is a common treatment in the past [18]. This method can control the type and concentration of antibiotics easily, and improve the bacterial clearance rate greatly. However, there are several disadvantages as follows: (1) The extravasation of flushing liquid and the blockage or fall of the drainage tube; (2) Long flushing time causes the patient to stay in bed for a long time; (3) The bacteria in the deep part of the lesion cannot be removed, and the remaining bacteria can easily cause the recurrence of the infection [19]. In this case, antibiotic artificial bone as a new treatment method was applied in the treatment of bone infection. Antibiotic artificial bone is a mixture of antibiotics and biomaterials with tissue compatibility and osteoconductivity, and then filled in the lesion to achieve the purpose of slow release of antibacterial drugs [20, 21]. The antibiotic carrier used in this study was calcium sulfate, which is a biological material that can be degraded in the human body. The antibiotic loaded with calcium sulfate could reach the release peak within 6~24 hours and maintain the antibacterial concentration for 30~60 days. The porous structure of calcium sulfate can make the antibiotic to penetrate sufficiently, it has more adequate elution of antibiotics after being absorbed absolutely in the human tissue. Calcium sulfate bone power can fill bone defects in the early stage and the later degradation process is also the process of new bone formation because of its good histocompatibility. It creats an opportunity for the repair of bone defects and speeds up the healing of bone defects [21, 22].

Due to the low incidence of sclerosing osteomyelitis, the current literatures are mostly case reports or small case series studies. Jamshidi analyzed retrospectively six patients with sclerosing osteomyelitis, of which four patients underwent surgery to scrape the infected tissue, and the recovery effect was better than 2 patients who did not underwent surgery [23]. Didier treated a 14-year-old child with sclerosing osteomyelitis only by surgical resection and no antibiotics were used after the operation which also achieved satisfactory results [4]. The researcher have reported the effectiveness of using ibandronate in the treatment of sclerosing osteomyelitis, and found that this drug can alleviate the pain of patients effectively, but this method has the risk of causing osteonecrosis in patients [3]. Compared to conservative treatment with drugs, patients with sclerosing osteomyelitis apply surgical resection timely can achieve better treatment results. In this study, we compared the effectiveness of FD and VCSI in the treatment of sclerosing osteomyelitis. Our research results showed that there was no significant difference in the amount of blood loss between the two surgical methods, but the operation time of the VCSI group was longer than FD group, because it took 15 minutes to wait for the drug to solidify during the preparation of vancomycin calcium sulfate particles. The results of the study showed that both the two surgical methods could relieve pain effectively, which was the most typical symptom of sclerosing osteomyelitis. Because two groups all received FD, this method could reduce the pressure in the medullary cavity and reduce the patient’s pain quickly. The postoperative hospital stay and the duration of drainage tube placement in the VCSI were lower than FD group. This was because patients underwent VCSI did not need to place a drainage tube for continuous flushing, which allowed the patient to move early and accelerates the patient’s recovery greatly. The vancomycin calcium sulfate implanted during the operation could release antibiotics for a certain period of time, which was conducive to the control of infection and the killing of local bacteria. However, calcium sulfate also has inherent weaknesses. The absorption of calcium sulfate can occur rapidly in the body, and the support strength it provides will fade with degradation. In addition, the degradation product of calcium sulfate is water, which will increase the drainage of the wound after surgery and aggravate the infection [21].

There are some limitations for this study. Firstly, a relatively low number of patients was included in the study. Secondly, the impact of different fixations (external fixation or not) on treatment results cannot be reliably judged due to the limited samples. Thirdly, the study is retrospective, which may be associated with some results bias. Therefore, it is necessary to carry out prospective large-sample randomized clinical controlled trials.

## Conclusion

Both FD and VCSI can relieve the pain of sclerosing osteomyelitis effectively. Compared with FD, although VCSI takes longer operation time, it has the following advantages: shorten the postoperative drainage tube placement time, shorten the postoperative hospital stay, controll the infection better.

## Data Availability

The datasets used and/or analysed during the current study are available from the corresponding author on reasonable request.
